# Supplementary figures and images for: Development of a social contact self-efficacy scale for ‘third agers’ in Japan
Source: PLoS One. 2021 Jun 22;16(6):e0253652. doi: 10.1371/journal.pone.0253652 (PMC8219158; doi:10.1371/journal.pone.0253652)

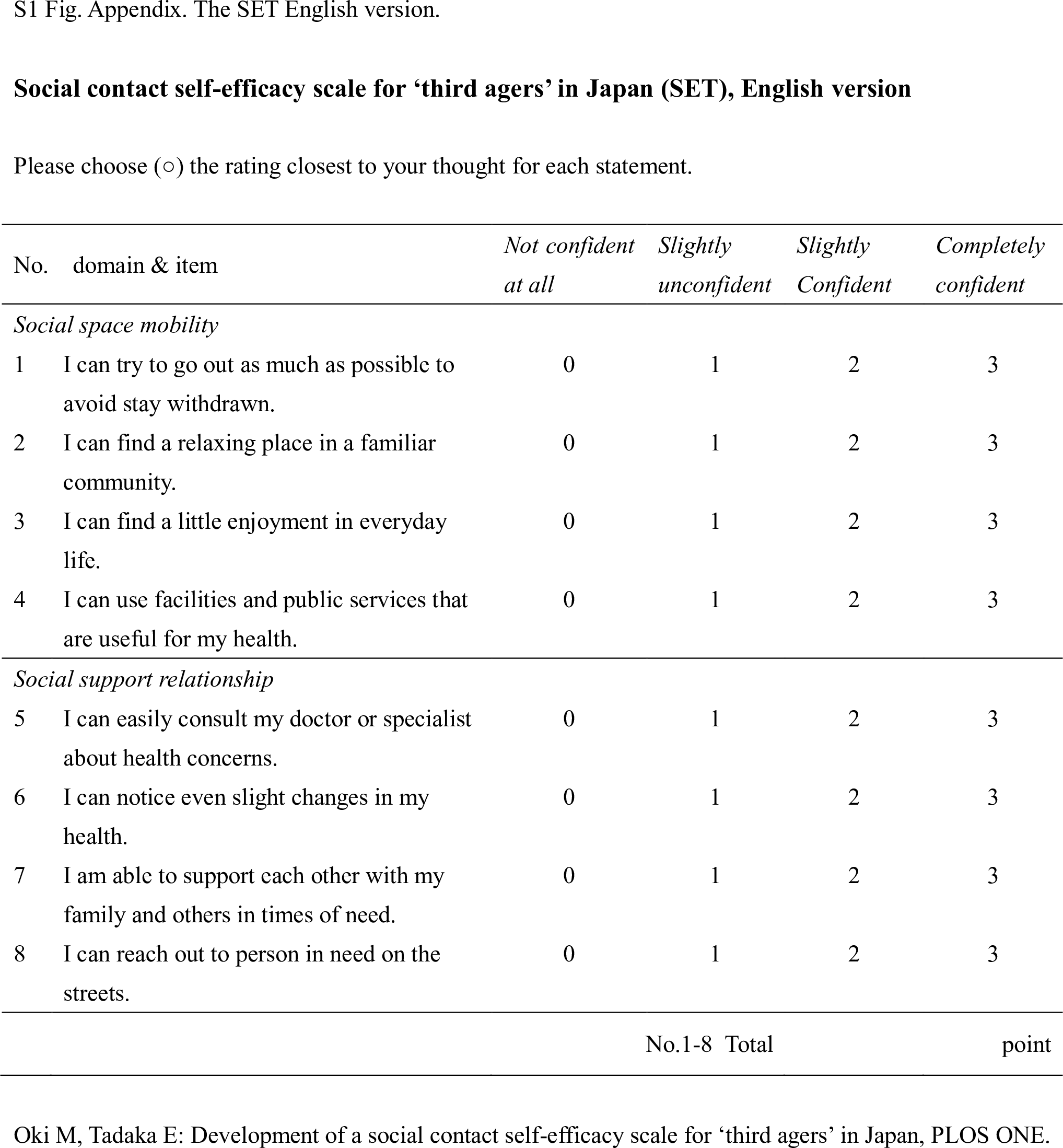

Supplement: S1 Fig — The SET English version. (TIF) [file pone.0253652.s001.tif]

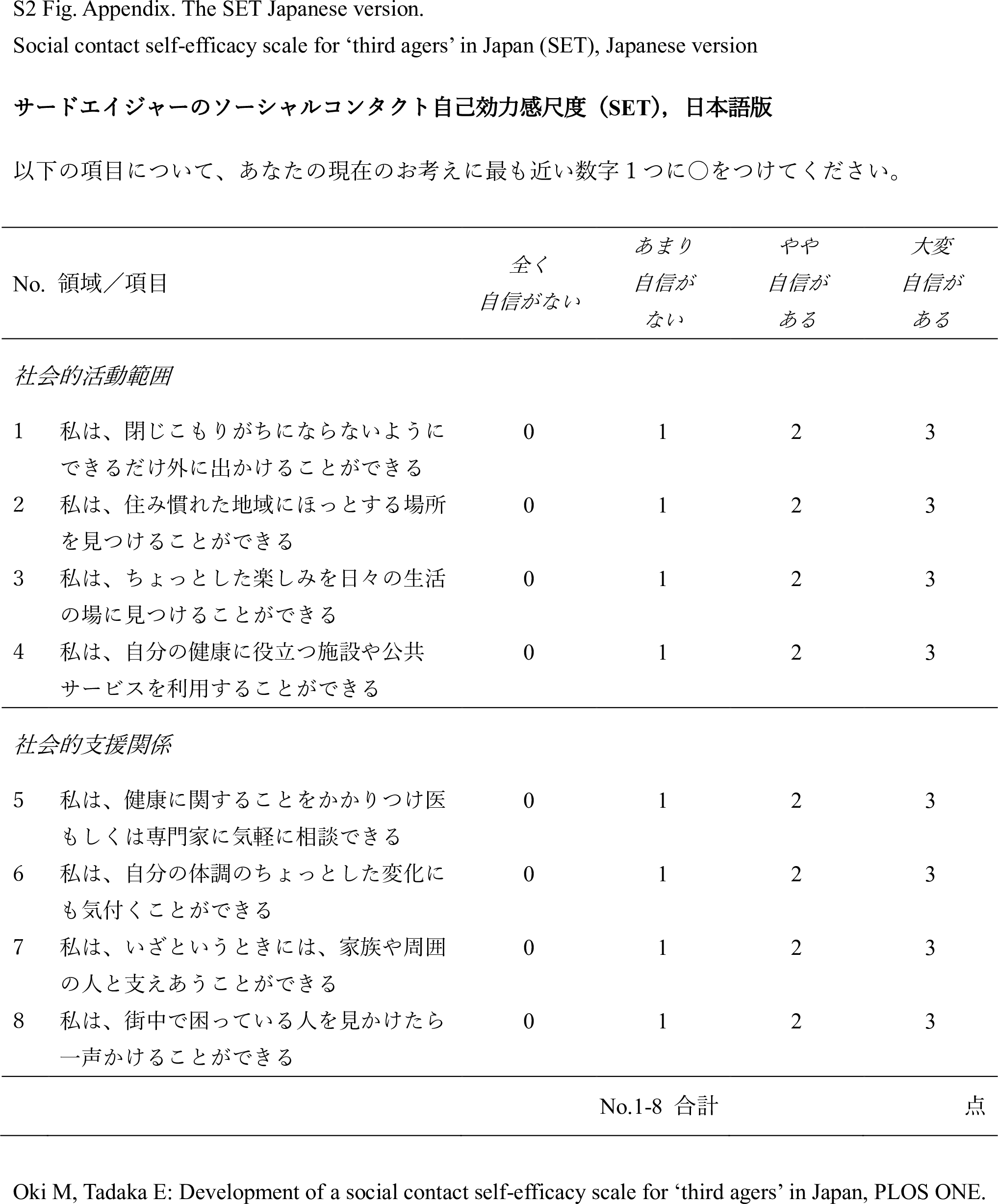

Supplement: S2 Fig — The SET Japanese version. (TIF) [file pone.0253652.s002.tif]
